# High-Risk HPVs and Human Carcinomas in the Syrian Population

**DOI:** 10.3389/fonc.2014.00068

**Published:** 2014-04-02

**Authors:** Ala-Eddin Al Moustafa, Lina Ghabreau, Nizar Akil, Samer Rastam, Amal Alachkar, Amber Yasmeen

**Affiliations:** ^1^ABS Research and Development, Montreal, QC, Canada; ^2^Oncology Department, McGill University, Montreal, QC, Canada; ^3^Department of Mechanical Engineering, Concordia University, Montreal, QC, Canada; ^4^Syrian Research Cancer Centre of the Syrian Society against Cancer, Aleppo, Syria; ^5^Faculty of Medicine, Pathology Department, Aleppo University, Aleppo, Syria; ^6^Faculty of Pharmacy, Pharmacology Department, Aleppo University, Aleppo, Syria

**Keywords:** HPVs, human carcinomas, Syrian population

## Abstract

Human papillomavirus (HPV) infection is the most common sexually transmitted infection; persistent infection with high-risk types of HPV present a major risk factor for the initiation and progression of a variety of human carcinomas including cervical, colorectal, head, and neck (HN) as well as breast carcinomas. A huge number of studies investigated and reported the incidence of high-risk HPVs in these cancers worldwide particularly in the developed countries; therefore, two HPV prophylactic vaccines against the two most frequent high-risk HPV types (16 and 18) have been developed and used worldwide. However, there are very limited studies about the prevalence of HPVs in the developing countries especially in Africa and some states of the Middle East. In this mini review, we outline the presence of high-risk HPVs in human cervical, colorectal, HN as well as breast cancers in the Syrian population, which was recently explored for the first time by a Canadian/Syrian group.

## Introduction

Human papillomaviruses (HPVs) are considered among the major viruses associated with human cancers especially cervical carcinomas (1). HPVs are the most common sexually transmitted infections worldwide, with the majority of individuals who engage in sexual activity becoming infected at some point in their lifetime; HPVs are small, double-stranded DNA viruses that generally infect cutaneous and mucosal epithelial tissues of the anogenital tract (1, 2). To date, over 120 different viral types have been identified; HPVs are classified as either high risk or low risk, with high-risk types being associated with cancer formation. Infections with low-risk types are generally self-limiting and do not lead to malignancy (3–5). However, infections with high-risk HPVs (type 16, 18, 31, 33, 35, 39, 45, 51, 52, 55, 56, 58, 59, 68, 73, 82, and 83) are associated with the development of cervical cancers where more than 96% of these cancers are positive for high-risk HPVs worldwide (1, 6). Moreover, accumulating evidence suggests that persistent infection with those viruses is necessary for cervical precursors to evolve into invasive carcinomas (1, 7, 8). High-risk HPVs are important risk factors for other human cancers as well, such as head and neck (HN) and colorectal carcinomas; as roughly 30 and 80% of these cancers are positive for high-risk HPVs, respectively (9–11). Moreover, it was observed that the presence of high-risk HPVs serve as a prognostic factor in early-stage cervical, HN, and colorectal cancers, and could be associated with vascular invasion, lymph node metastases, and tumor size (12–16). In addition, numerous recent studies revealed that high-risk HPVs are present in human breast cancers worldwide (17–21); controversially few studies did not confirm this statement (22–24).

Earlier studies revealed that the prevalence of HPV infections in human cancers is related to specific geographic locations worldwide (3, 25). On the other hand, it is important to mention that the majority of studies on the incidence of HPVs and their role in human cancers were carried out in countries with high to intermediate economic rank (25); however, there are a small number of investigations regarding the presence and incidence of these viruses in the developing countries including Africa and some states of the Middle East (ME) (3, 26). Thus in this paper, we reviewed cancer cases identified by the Pathology Department of the main teaching hospital of the Faculty of Medicine of Aleppo University, which is one of the major universities in Syria, and investigated the presence and distribution of high-risk HPVs in cervical, HN, colorectal, and breast cancers in the Syrian population.

## High-Risk HPVs in Cervical Cancer

It is well established that high-risk HPVs are important risk factors for human cervical cancers; as roughly 96% of these cancers are positive for high-risk HPVs, and the most frequent HPV types are 16 and 18 worldwide (6, 7); therefore, two HPV vaccines against these two types of viruses have been generated and spread worldwide (Gardasil^®^) and (Cervarix^®^). Meanwhile, it is important to emphasize that there are few studies regarding the presence and distribution of high-risk HPVs in human cervical cancers in the majority of the developing countries (26, 27), which is an important initiator to HPV vaccines introduction in these countries. Accordingly, there is only one study about the incidence of high-risk HPVs in cervical cancer on the Syrian population, which was recently performed in collaboration between a Canadian group of McGill University and their colleagues of Aleppo University (28). In this study, the authors investigated the presence of high-risk HPV types 16, 18, 31, 33 35, 45, 51, 52, and 58 in a cohort of 44 cervical cancer samples from Syrian women, with an average age of 57 years, by polymerase chain reaction (PCR) analysis, using specific primers for E7 gene, and immunohistochemistry (IHC) methodology (Table 1). This study revealed that 38 (95.45%) of the 42 samples are HPV positive and all of these positive specimens are co-infected with more than one HPV type; moreover, HPV types 33, 16, 18, 45, and 52 are the predominant viruses of the high-risk HPVs family in these cervical cancer tissues. On the other hand, this investigation reported that the presence of high-risk HPVs is correlated with Id-1 gene over-expression in 95.23% of human cervical cancers, which are invasive carcinomas in the majority of cases. Accordingly, it was displayed that Id-1, a member of the helix–loop–helix transcription factor family, is expressed in the majority of invasive cervical carcinomas (29). More significantly, this study revealed that the prevalence of high-risk HPV in carcinomas of the cervix in Syria is approximately similar to those reported in other countries in the ME and North Africa, particularly in Turkey (30–53). Nevertheless, we believe that future studies on a larger number of samples, from several provinces of Syria are needed to confirm the incidence of HPVs in cervical cancer in this country.

**Table 1:** **List of studies regarding the incidence of high-risk HPVs in human cervical, head and neck, colorectal, and breast cancer in the Syrian population**.

Anatomical cancer site	Number of cases	Assay	HPVs positive %	Most frequent HPV types	Reference
Cervical	44	PCR/TM^a^	95.45	33, 16, 18, 45, and 52	Darnel et al. (28)
Head and neck	80	PCR/TM	43	16, 18, 31, 33, and 35^b^	In preparation
Colorectal	78	PCR/TM	53.84	16, 33, 18, 35, and 31	Ghabreau et al. (69)
Breast	113	PCR/TM	61.06	33, 35, 16, 81, and 51	Akil et al. (75)

*^a^Tissue microarray*.

*^b^These data are preliminary*.

*All these studies were done on paraffin-embedded tissue samples that were collected from the major teaching*.

*Hospital of the Faculty of Medicine of Aleppo University*.

## High-Risk HPVs in Head and Neck Cancers

Cancers of the HN (upper aerodigestive tract) include neoplasias of the oral cavity, the pharynx (naso-, oro-, and hypopharynx), the larynx, and the paranasal sinuses (54). Head and neck squamous cell carcinomas (HNSCCs) are the predominant tumors of the HN comprising more than 95% of all HN cancers (55, 56). Currently it is assumed that high-risk HPVs infections are important factors in the development of HNSCCs, as approximately 30% of these cancers are positive for high-risk HPVs (9, 10, 57). As we mentioned above, the presence and allocation of these viruses in human cancers, including HNSCCs, are related to specific geographic locations worldwide (3, 10, 25). However, the presence of HPVs in HN cancers in the developing countries, including Syria, is limited to insufficient number of studies. For instance in Syria, the same Canadian/Syrian group recently investigated the presence of high-risk HPVs in a cohort of 80 HN cancer tissue samples (7 females and 73 males with a median age of 54.5 years) from the Syrian population using IHC analysis and tissue microarray methodology. All the samples were squamous cell carcinomas (57 larynx, 21 lips, and 1 nasopharynx). The data of this study revealed that 43% of these cancers are positives for high-risk HPVs. Genotyping of high-risk HPVs is presently under investigation by the same group (Table 1); however, preliminary data from this study reveal that HPV types 16, 18, 31, 33, and 35 are frequent in HN cancers in Syria (Ghabreau et al., in preparation). On the other hand, authors of this investigation explored the association between the presence of high-risk HPVs and p53 expression in this population; their data did not show an evident correlation between these viruses and p53 in their samples.

Accordingly, the incidence of high-risk HPVs in HN cancers in the Syrian population is comparable to the prevalence of these viruses in Turkey. It is worth mentioning that Turkey is one of the neighboring countries of Syria where HPV prevalence studies have been conducted in HN; specifically, there have been four studies exploring the presence and distribution of HPVs in HN cancers using PCR methodology; these studies showed that the presence of high-risk HPVs varies from 14 to 47.6%, and the most frequent HPVs in the Turkish population are HPV types 16, 18, 31, and 33 (58–60). However, there have not been any similar studies to date in the other neighboring countries such as Iraq, Lebanon, and Jordan with the exception of one study conducted in Israel. In this investigation, the authors examined the presence of only HPV type 16 in 23 oral and oropharynx carcinoma cases by PCR; their data revealed that four samples (17.3%) are positive for this type of high-risk HPV (61).

In conclusion, it is evident that high-risk HPVs are present in HN cancers in the Syrian population and their presence is comparable to HN cancers in Turkey. However, it is important to highlight that the Canadian/Syrian study was limited to an insignificant number of cases located in a single region of Syria; therefore, it is essential to perform other studies to confirm the incidence and types of these viruses in the Syrian population.

## High-Risk HPVs in Colorectal Cancer

Colorectal cancer is the third most common type of cancer, with approximately 1.3 million new cases diagnosed annually worldwide, accounting for about 9.7% of all cancer cases (WHO). Evidence from recent studies suggests that environmental conditions and lifestyle in addition to sequential genetic changes and possibly viral components are major risk factors for colorectal cancer (62). Therefore, it was recently pointed-out that high-risk HPVs have carcinogenic effects at several anatomical sites in both women and men such as colorectal (16, 63, 64). These studies showed that high-risk HPVs are present in approximately 80% of colorectal cancers, especially in their invasive form worldwide; however, a small number of studies, including ones from ME (65), did not confirm this fact. In this context, it is important to highlight that there is a few investigations about the presence of HPVs in colorectal cancers in the developing countries including one study from Syria, which was performed by the Canadian/Syrian group (Table 1). This group has investigated the presence of high-risk HPVs and their association with Fascin, Id-1, and P-cadherin genes, which are major regulators of cell invasion and metastasis (66–68), in human colorectal cancers in the Syrian population (69). In this study, the authors used PCR and tissue microarray analysis to explore the presence of HPV and E6 expression, respectively, in a cohort of 78 cancer tissues (41 females and 37 males with a median age of 49 years). This study revealed that high-risk HPVs are present in 42 samples (53.84%); the most frequent high-risk HPV types in the Syrian population are 16, 33, 18, 35, and 31, respectively. Furthermore, the expression of E6 onco-protein of high-risk HPVs was found to be associated with Fascin, Id-1, and P-cadherin expression in the majority of cancer tissue samples. Data of this investigation showed, for the first time, that high-risk HPVs are present in human colorectal cancers in the Syrian population and their presence is associated with invasive and metastatic phenotype (69). On the other hand, it is important to indicate that the presence of HPVs in colorectal cancers in the ME is limited only to four studies one from Israel (65) and three from Turkey (70–72). The first study, from Turkey, found that high-risk HPVs are present in 46 of 56 colorectal cancer tissues, which represent 82.14% HPV-positive cases (70). This study revealed that the most frequent HPVs in colorectal cancers in Turkey are HPV types 18 and 33. The second study was conducted on a cohort of 43 colorectal cancer tissues, and reported that 55.8% of these cancer cases are positive for high-risk HPVs especially types 18 and 33 (71). However, the third study was unable to detect the presence of HPVs in 106 colorectal cancer samples (72). These studies used PCR approach to detect the presence of HPVs in their samples. In conclusion, the Syrian and Turkish studies suggest that HPV types 18 and 33 are common in colorectal cancers in Syria as well as Turkey; in this context, it is important to mention that the Syrian study was conducted in Aleppo, which is close to the Turkish border. However, we believe that more investigations in several areas of Syria are necessary to elicit a clear image of the presence and distribution of high-risk HPVs in colorectal cancers in the Syrian population.

## High-Risk HPVs in Breast Cancer

Breast cancer is the most common malignancy in women worldwide; and metastatic breast disease is a major cause of morbidity and mortality in breast cancer patients. Several earlier studies reported that high-risk HPVs are present in approximately 50% of human breast cancers worldwide (17–21); controversially a few studies were unable to detect the presence of HPVs in breast cancer as well as normal mammary tissues (22–24). On the other hand, it was pointed-out that the presence of high-risk HPVs especially types 16 and 18, in human breast cancer, is correlated with invasive carcinomas phenotype (19, 21, 73). More significantly, it was demonstrated that E6/E7 onco-proteins of HPV type 16 covert non-invasive and non-metastatic breast cancer cells to invasive and metastatic form, *in vitro* and in nude mice, respectively (3, 74). Regarding the presence of high-risk HPVs in breast cancer in Syrian women, the Canadian/Syrian group investigated the incidence of HPV types 16, 18, 31, 33, and 35 in a cohort of 113 breast cancer tissue samples (with a median age of 52 years) by PCR analysis using specific primers for their E6 and/or E7 genes and tissue microarray approach (75). This study revealed that 69 (61.06%) of the 113 samples are HPV positive and 24 (34.78%) are co-infected with more than one HPV type; in addition, HPV types 16, 18, and 31 are present in 10, 11, and 8 cancer tissues, respectively. In contrast, 63 and 42 cancer tissues were positive for HPV types 33 and 35, respectively. Therefore, the authors concluded that the most frequent high-risk HPVs in breast cancer in Syrian women are HPV types 33 and 35. On the other hand, this investigation reported that the presence of high-risk HPVs is correlated with Id-1 gene over-expression in human breast cancers, as it was revealed in human cervical cancer (28).

The Canadian/Syrian group data of HPVs in breast cancers (Table 1) are similar to a Turkish study, which was performed in 50 breast cancers and normal mammary tissues using PCR analysis (76). In this study, authors reported that 74% of malignant breast tissues are positive for HPVs; and the most common high-risk HPVs in the Turkish women are types 33 and 35. Herein, it is important to mention that there is another study from ME (from Tunisia) that did not confirm the presence of HPVs in breast cancer (24). In conclusion, we believe that HPVs are present in human breast cancer in the ME region including Syria. However, more investigations are necessary to confirm their present and distribution of HPVs in the Syrian population.

## Conclusion and Future Perspective

In this paper, we have reviewed the incidence of high-risk HPV infections and their associated cancers including cervical, HN, colorectal, and breast in the Syrian population. Although data of HPVs and their related cancers worldwide are evident, the incidence of HPVs in the Syrian population needs more investigations to be confirmed. The presence of these viruses in Syria is limited to one study from the same group for each anatomical site; in addition, these studies are conducted in one region of Syria, which is near the Turkish border. It is important to highlight that presently available HPVs vaccines can protect only against HPV types 16 and 18 and their associated cancers, which are the most frequent types in western countries; so far this does not seem to be the case in the Syrian population based on the Canadian/Syrian group studies [(77) and Table 1]. In addition and based on the cost and efficacy of the two available vaccines as well as the availability of the second generation of HPV vaccine, which will be against high-risk types 16, 18, 31, 33, 45, 52, and 58, in addition to two low-risk types, 6 and 11 (78), we believe that the populations of developing countries including Syria should wait for the new generation of the HPV’s vaccine. Meanwhile, we incite our colleagues in developing countries including Syria to reassess the incidence of high-risk HPVs in their respective populations, which is an important step to eliminate these viruses and associated cancers worldwide.

## Conflict of Interest Statement

The authors declare that the research was conducted in the absence of any commercial or financial relationships that could be construed as a potential conflict of interest.
